# Harnessing gut-derived bioactives and AI diagnostics for the next generation of type 2 diabetes solutions

**DOI:** 10.3389/fendo.2025.1699954

**Published:** 2025-11-03

**Authors:** Yuliya Tseyslyer, Vladyslav Malyi, Mariia Saifullina, Olena Tsyryuk, Yuliia Shvets, Yurii Penchuk, Iryna Kovalchuk, Oleksandr Kovalchuk, Oleksandr Korotkyi, Volodymyr Bulda, Olena Lazarieva

**Affiliations:** ^1^ Educational and Scientific Centre, Institute of Biology and Medicine, Taras Shevchenko National University of Kyiv, Kyiv, Ukraine; ^2^ Department of Therapy and Rehabilitation, National University of Ukraine on Physical Education and Sport, Kyiv, Ukraine; ^3^ Department of Normal Physiology, State Non-Profit Enterprise “Danylo Halytsky Lviv National Medical University”, Lviv, Ukraine

**Keywords:** type 2 diabetes, artificial intelligence, microbiome, gut microbiota, digital twin systems, deep learning models, closed-loop systems

## Abstract

**Introduction:**

The prevalence of type 2 diabetes (T2D) has significantly increased over the past 20 years, currently affecting over 500 million people worldwide. Projections suggest that this number could rise to over 700 million in the next two decades. Despite advancements in medication and global health strategies that promote healthy lifestyles, T2D remains a complex disease that impacts the quality of life. Traditional treatment methods are becoming less effective, highlighting the need for innovative approaches to prevention, diagnosis, and treatment.

**Methods:**

Two promising areas of research that could transform the management of T2D are the use of biologically active substances derived from the intestines and the integration of artificial intelligence (AI) in clinical diagnostics. The human intestinal microbiota plays a crucial role in metabolic processes, including glucose regulation and insulin sensitivity. Microbial metabolites, including bile acids and short-chain fatty acids, have potential as therapeutic agents for metabolic disorders. As digital medicine advances, AI is increasingly utilized for real-time monitoring and personalized risk assessments. The medical field is evolving from merely using biosensors for glucose tracking to employing machine learning to analyze various biological indicators and electronic medical records.

**Results:**

Recent research at the intersection of microbiome studies and AI may improve diagnostic accuracy and support tailored treatment strategies. This study aims to analyze global experiences with the implementation of bioactive substances from the intestines and the diagnostic potential of AI in developing a new approach to enhancing the quality of life and treating T2D.

**Discussion:**

We examine the diverse functions of microbial metabolites and the current landscape of their therapeutic applications. Additionally, the review examines the current state of AI in diagnostics, with a particular focus on microbiome parameters. As a result, we propose a novel model that combines these two fields into an adaptive and personalized approach to treating patients with T2D and improving their quality of life.

## 1 Introduction

### 1.1 Burden of type 2 diabetes and obesity

The progress of civilization has brought remarkable achievements in technology, modernization, healthcare, urbanization and accelerated socio-economic development, ultimately enhanced living standards worldwide. However, it has also affected daily living patterns, particularly eating habits and levels of physical activity. Sedentary lifestyles, widespread availability of energy-dense processed foods and unhealthy eating habits have driven the growth of obesity across the globe. Within the past twenty years, obesity has become a global pandemic that negatively affects health, impacting almost all organs of the human body (1–4).

Statistics indicate that between August 2021 and August 2023, the prevalence of obesity among adults in the United States was 40.3%, with no significant differences between men and women. Obesity was more common in adults aged 40–59 years than in those aged 20–39 years or ≥60 years (5). A systematic review and meta-analysis in Africa reported that 61.4% of adults are overweight or obese, with prevalence ranging from 56.9% in East Africa to 88.5% in Southern Africa (6). A study in the Middle East reported that 85.8% of individuals with T2D were classified as overweight or obese (7). A recent meta-analysis (8) reported that 75.27% of pediatric patients with T2D were obese, and 77.24% were obese at diagnosis; obesity prevalence was higher among males compared to females, and highest in North America.

A marked increase in obesity across all age groups has become one of the main factors driving the rapid rise in cases of T2D. Obesity has become a significant public health concern, now ranking among the most common non-communicable diseases. As body mass index (BMI) increases across different age groups, the risk of developing T2D rises proportionally. Recent data indicate that the incidence of T2D is rising rapidly among younger populations. The results of numerous studies show that obesity contributes to an increased risk of several long-term health disorders. These include cardiovascular diseases, metabolic syndrome, type 2 diabetes mellitus (T2D), chronic kidney disease, and various malignancies (9–11). This burden of T2D and obesity underscores the need to understand its underlying molecular mechanisms for developing innovative strategies.

Obesity has become a significant public health concern, now ranking among the most common non-communicable diseases. As body mass index (BMI) increases across different age groups, the risk of developing type T2D rises proportionally. Recent data indicate that the incidence of T2D is rising rapidly among younger populations (12, 13).

### 1.2 Potential molecular mechanisms in T2D pathogenesis

T2D and obesity are connected through shared mechanisms such as chronic low-grade inflammation, β-cell dysfunction, and insulin resistance (IR) (14, 15). Visceral obesity, defined by excess adipose tissue around internal organs, induces a chronic inflammatory process that disrupts metabolism and promotes IR. The accumulation of visceral fat affects the rate of lipolysis and increases the release of free fatty acids into the portal circulation, which subsequently modifies liver metabolism and further contributes to IR (16, 17). Chronic obesity induces a prolonged inflammatory response that leads to tissue fibrosis and irreversible organ damage, thereby contributing to multiple organ dysfunction (18). This inflammatory state results from altered immune cell function in adipose tissue, skeletal muscle, and liver. As a result, conditions that promote the development of IR arise, leading to an increased risk of T2D (19, 20).

The inflammatory response activates inflammasome complexes, specifically the NLRP3 inflammasome (21), which promote the maturation and release of pro-inflammatory cytokines, particularly interleukins IL-1β and IL-18. These mechanisms can provoke the development of IR, which, in turn, contributes to the onset of T2D (22). IR is considered a key cause of T2D. The accumulation and activation of pro-inflammatory macrophages in adipose tissue is a key driver of chronic low-grade inflammation. An increased number of these macrophages produces factors that act in a paracrine or systemic manner, disrupting insulin signaling in target cells (14, 23).

Currently, a confirmed association exists between metabolic disorders and the gut microbiota (24, 25). Gut microbiota dysbiosis is known to trigger inflammatory processes that impair glucose tolerance and promote IR (26). Although current studies present differing views on whether obesity or inflammatory bowel diseases are primary condition, intestinal inflammation can impair digestive processes and contribute to obesity. Conversely, poor nutrition and disturbances in intestinal homeostasis in overweight individuals can alter gut microbiota, leading to dysbiosis, which is considered one of the main causes of inflammation (24). Diet is a major factor influencing the gut microbiome and can lead to dysbiosis. Research has proven that disruption of mucosal barrier integrity enables microbial signaling that activates the nuclear transcription factor kappa B (NF-kB) and stimulates the production of pro-inflammatory cytokines. In adipose tissue, which contains both adipocytes and macrophages, the induction of monocyte chemoattractant protein-1 (MCP-1) expression promotes macrophage infiltration into tissues (24).

The composition of gut microbiota plays a key role in obesity, metabolic syndrome and T2D by contributing to decreased glucose tolerance and IR. Alterations in gut microbiota composition have been observed in preclinical animal models as well as in T2D patients with associated complications, such as diabetic neuropathy, nephropathy, osteoarthritis, retinopathy, cerebrovascular and cardiovascular diseases, compared to healthy control participants. The degree of gut microbiota dysbiosis correlated with disease severity, and its restoration, achieved through probiotic administration in both animals and human patients, demonstrated improvement in symptoms and slowing of disease progression (26–32). It is known that certain medications, notably metformin, which is commonly used to treat T2D, affect gut microbiota composition, demonstrating interaction with the intestinal microbiota through modulation of inflammatory processes, regulation of glucose homeostasis, influence on intestinal barrier permeability, and promotion of short-chain fatty acid–producing bacteria (33–35).

Over the past decade, rapid advancements in microbial genome sequencing technologies have triggered a wave of research investigating the involvement of gut microbiota in various pathologies, especially metabolic conditions. Although significant progress has been made in understanding the complex interactions between bacteria and the host organism, how gut bacteria directly influence the prevention, development, or treatment of diseases remains an active area of scientific investigation. This is especially relevant in many pathologies where the impact of the gut microbiota has been studied, including dysbiosis and its role in the development of T2D and related complications. Current trends in the study of bioactive substances produced by the microbiota open new opportunities for both the prevention and individualized treatment of metabolic diseases. These advances have driven the integration of biomedical data with artificial intelligence (AI), which not only allows more efficient analysis of large volumes of genomic, metabolomic, and clinical information but also offers prospects for early prediction of T2D development, risk stratification, and optimization of therapeutic strategies. These mechanisms pave the way for innovative approaches, such as utilizing gut-derived bioactive substances and AI for diagnosis and treatment.

### 1.3 Aim and perspectives

The aim of this article is to evaluate current evidence on the involvement of gut microbiota and its bioactive metabolites in T2D, as well as to review modern possibilities for using artificial intelligence for early diagnosis, prediction of complications, and personalized treatment approaches in patients with T2D. Special attention is given to the interplay between the metabolic activity of the microbiota and innovative AI-based diagnostic technologies, which have the potential to transform the traditional therapeutic paradigm for managing this disease.

## 2 Methods

This review article summarizes the current evidence regarding the role of gut microbiota and its bioactive metabolites in T2D. It also explores the applications of AI for early diagnosis, risk prediction, and personalized treatment of the condition. To achieve this, we adopted a narrative review approach that incorporates elements of systematic searching, ensuring comprehensive coverage of the topic. This methodology was selected to integrate a diverse range of biomedical data, including genomic, metabolomic, and clinical information, while considering the interdisciplinary nature of the subject. By doing so, we aim to evaluate the evidence, examine the possibilities for AI integration, and analyze the interactions between microbiota activity and diagnostic technologies.

## 3 Results

### 3.1 Human microbiota

Recently, the human microbiota has become a focal point in many biomedical studies (36, 37). It includes all microorganisms residing in the body, including bacteria, microscopic fungi, protozoa, viruses, and archaea (38). Among the various microorganisms, research has predominantly concentrated on those within the domain *Bacteria*. It is now established that approximately 15,000 bacterial species inhabit the human species *Homo sapiens* (39). The microbiota of an individual typically contains around 1000 bacterial species (40). Greater species diversity in the microbiota is frequently associated with improved health outcomes (41).

Early studies of the microbiota revealed a complex and interdependent relationship between the host and its microbial communities. The macroorganism provides the microbiota with a comfortable ecological niche and nutrients, while the microbiota, directly or indirectly, influences the functioning of all organs and systems throughout the body. This dynamic interaction ultimately determines overall health, susceptibility to disease, and the effectiveness of response to treatment and vaccination (42, 43).

The human gut microbiota is known to be the most numerous microbial community in the body and, consequently, exerts a significant impact on health. Based on the composition of microbial associations and the characteristics of host organism’s response, this influence can be both positive and negative. Ilya Mechnikov, the Ukrainian researcher and Nobel laureate, was among the first to highlight this relationship. He noted that improving the microbiota of the large intestine through the consumption of fermented dairy products has a health-promoting effect and may even contribute to longevity (44).

Since the appearance and development of the holobiont concept, there has been a growing understanding of the human organism as a superorganism — a complex ecological system inhabited by hundreds of microbial species (45). The microbiota is thus considered an additional metabolic organ, whose activity is integrated into the functioning of various organs and systems of the macroorganism (46). Consequently, disruptions in the healthy composition and metabolic activity of the microbiota are observed in a variety human diseases, including gastrointestinal, cardiovascular, and endocrine pathologies, immune and nervous system dysfunctions, and various cancers (47, 48).

As noted above, bacterial microbiota is currently the most extensively studied. It is well known that different bacterial species possess unique metabolic properties that contribute to the overall metabolism of the human organism. It becomes clear that in pathological conditions, changes in the microbiota reflect and impact the metabolism of the macroorganism.

Several pathological conditions, including metabolic syndrome, obesity, T2D, non-alcoholic fatty liver disease, and cardiometabolic syndrome, are linked to metabolic disturbances. Dysbiotic changes in the gut microbiota occur in all these conditions (49–51).

#### 3.1.1 Gut microbiota composition in T2D: general overview

One of the most common metabolic disorders in older adults is T2D. Most patients with T2D are overweight and demonstrate IR in multiple tissues, including muscle, liver, and adipose tissue, during the early stages of the disease, which leads to a compensatory increase in insulin production. These patients commonly present not only with hyperglycemia but also with elevated triglycerides and low-density lipoproteins, hypertension, enhanced platelet adhesiveness and impaired microcirculation. All these factors contribute to comorbidities such as neuropathies, nephropathies and retinopathies, with atherosclerosis being one of the most serious complications of T2D. Therefore, multidrug therapy is frequently necessary to manage blood glucose and reduce cardiovascular risk.

Multiple studies have shown that patients with T2D demonstrate significant differences in gut microbiota composition and metabolic activity compared with healthy individuals (52–54). Thus, T2D develops against the background of intestinal dysbiosis, which forms the basis for this metabolic disease. Typically, there is a shift in bacterial populations, with a rise in pro-inflammatory species and a reduction in bacteria that produce beneficial metabolites. The overall diversity of bacterial species in T2D also reduced. These microbiota modifications contribute to the disruption of glucose and lipid metabolism in affected individuals.

**Figure 1:**
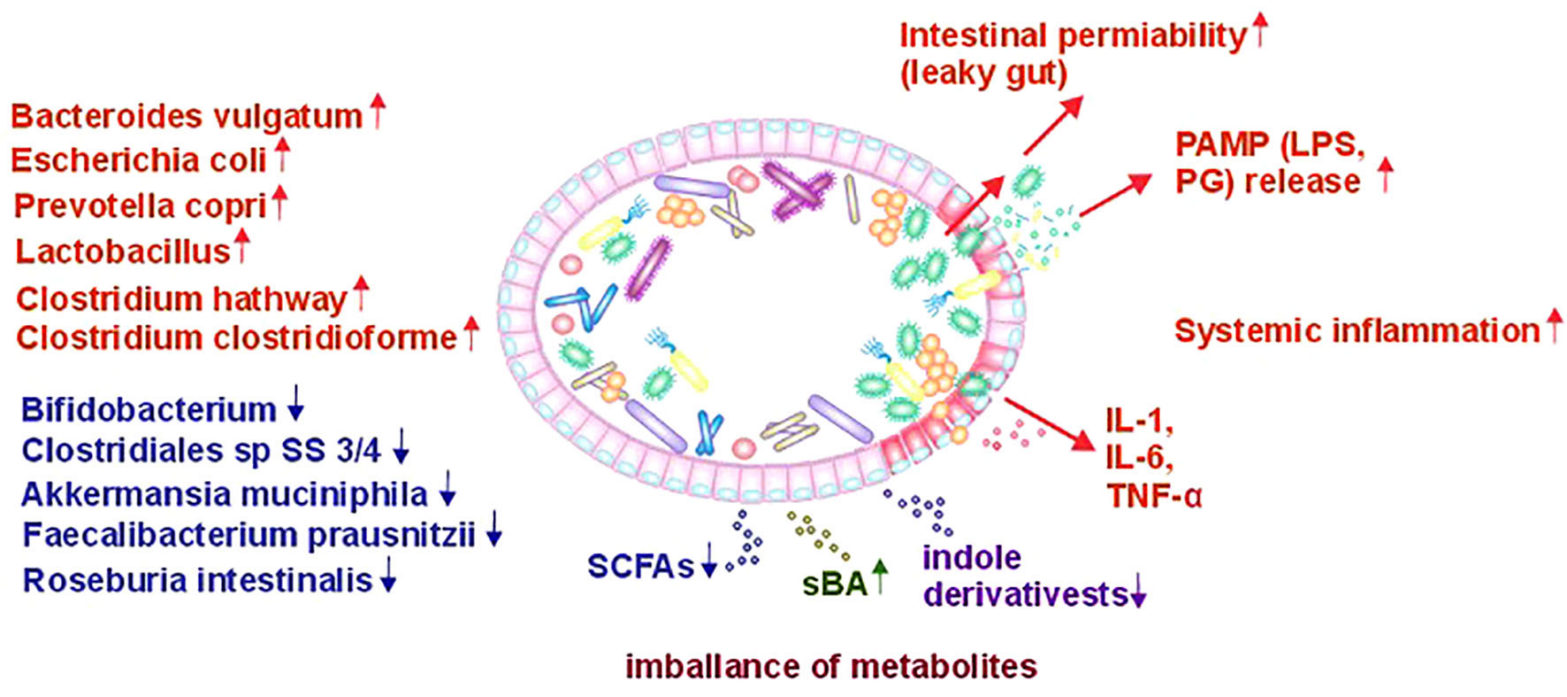
Role of gut microbiota alterations in the pathophysiology of T2D (PAMP, Pathogen Associated Molecular Pattern; LPS, Lipopolysaccharide; PG, Peptidoglycan; SCFA, Short Chain Fatty Acids; IL, Interleukin; TNF-α, Tumor Necrosis Factor-α).

Studies have shown that individuals with prediabetes have lower abundances of butyrate-producing bacteria, including the genera *Roseburia* and *Faecalibacterium*, as well as *Akkermansia muciniphila*. Instead, the microbiota has an increased content of opportunistic bacteria with pro-inflammatory properties (55, 56) (**Figure 1**).

Multiple studies of gut microbiota in European and Chinese populations have shown that patients with T2D have lower levels of butyrate-producing bacteria, including *Roseburia intestinalis* and *Faecalibacterium prausnitzii*, while showing increased abundances of *Lactobacillus* species and opportunistic bacteria belonging to *Bacteroides caccae*, *Clostridium hathewayi*, *Clostridium ramosum*, *Clostridium symbiosum* and *Escherichia coli* (57–59). Authors of one study (59) noted that the quantity of *Lactobacillus* were positively associated with fasting glucose and glycosylated hemoglobin (HbA1c) levels, while *Clostridium* species correlated negatively with these markers and plasma triglycerides, highlighting a potential role in T2D development. Newly diagnosed T2D patients showed higher levels of *Lactobacillu*s and lower levels of *Clostridium coccoides* and *Clostridium leptum*. Another study involving the Henan Rural Cohort population reported that the genera *Prevotella_9* and *Odoribacter* were inversely correlated with T2D, while the quantity of bacteria from the genus *Blautia* species were positively associated with the disease (60). Notably, all three of these bacterial genera produce of short-chain fatty acids (SCFAs). However, *Odoribacter* species predominantly produce butyric and acetic acids, *Prevotella* species mainly produce propionic and acetic acids, and *Blautia* species primarily produce acetic acid, with some species also capable of producing butyric acid. Therefore, when discussing SCFAs, produced by the entire gut bacterial community, the total amount and relative proportions of these acids in the gut and peripheral blood are critically important. In a healthy organism, the molar ratio of these acids (acetate: propionate: butyrate) is approximately 3:1:1 (61). During the development of T2D, overall SCFA levels are reduced, accompanied by a shift in their distribution, with lower amounts of butyrate and propionate and a higher proportion of acetate (60).

#### 3.1.2 Mechanisms of gut microbiota influence on T2D development

The gut microbiota exerts complex and multifactorial effects on the human body. However, in our view, the role of the microbiota in T2D can be summarized through several key mechanisms. Firstly, commensal gut bacteria produce a variety of metabolites, including SCFAs, aromatic and branched-chain amino acids, secondary bile acids (sBAs), tryptophan and indole derivatives, and trimethylamine-N-oxide (TMAO), which play roles in the disease’s pathogenesis (62). Under dysbiotic conditions, the normal composition of the gut microbiota is disrupted, resulting in significant changes in its metabolic activity. Studies in patients with T2D indicate that the gut microbiota is enriched in pathways such as sugar transport across membranes, which increases glucose uptake by cells; excretion of branched-chain amino acids (BCAA), contributing to IR; methane metabolism associated with the anaerobic gut environment; xenobiotic degradation; metabolic transformations linked to drug resistance; and sulfate reduction, which decreases insulin sensitivity. Ongoing research continues to focus on identifying microbiota-derived metabolite changes that could serve as biomarkers for susceptibility to various diseases, including T2D (63). A summary of key bioactive metabolites and their roles is provided in **Table 1**.

**Table 1:** The production of metabolites by gut microbiota.

Bacterial metabolites		Bacterial species	References
Short chain fatty acids	Acetic acid	*Prevotella* spp.*, Bifidobacterium* spp.*, Bacteroides* spp.*, Akkermansia muciniphila, Clostridium* spp.*, Streptococcus* spp.*, Ruminococcus* spp., *Blautia hydrogenotrophica*	(64–66)
	Propionic acid	*Bacteroides* spp.*, Megasphaera elsdenii, Veillonella* spp.*, Coprococcus catus, Salmonella* spp.*, Akkermansia muciniphila, Phascolarctobacterium succinatutens*, *Dialister* spp., *Roseburia inulinivorans*, *Blautia obeum*	
	Butyric acid	*Coprococcus comes, C. catus, C. eutactus, Faecalibacterium prausnitzii, Eubacterium hallii, Ruminococcus bromii, Eubacterium rectale, Anaerostipes* spp.	
Bile acid metabolites	Bile acid deconjugation	*Clostridium* spp.*, Bifidobacterium* spp., *Enterococcus* spp., *Lactobacillus* spp., *Bacteroides* spp., *Methanobrevibacter smithii, Methanosphera stadmanae*	(52, 66, 67)
	Secondary bile acid production	*Clostridium* spp.*, Eubacterium* spp.	
Tryptophan metabolites		*Escherichia* spp., *Proteus* spp., *Bacteroides* spp., *Clostridium* spp., *Peptostreptococcus* spp., *Lactobacillus* spp., *Enterococcus* spp., *Eubacterium* spp., *Anaerostipes* spp., *Bifidobacterium* spp.	(68, 69)

Secondly, under conditions of gut dysbiosis in T2D, the microbiota shows an elevated presence of opportunistic pro-inflammatory bacteria, initiating local intestinal inflammation. This inflammation underlies a third mechanism - increased intestinal wall permeability, often referred to as the ‘leaky gut’ phenomenon. As a result, various substances, known as pathogen-associated molecular patterns (PAMPs), which possess pro-inflammatory properties and are components of bacterial cells, primarily bacterial cell walls such as peptidoglycans (PG) and lipopolysaccharides (LPS), penetrate the intestinal mucosal barrier more easily and enter the bloodstream. This results in pro-inflammatory activation of vascular endothelial cells (70, 71) and peripheral immune cells, which is generally a sign of systemic inflammation (72, 73). Inflammation is a key precursor to metabolic syndrome, increasing risks of hypertension, visceral obesity, and dyslipidemia, which can damage pancreatic β-cells and reduce insulin secretion, contributing to T2D. While there is evidence showing the beneficial effects of probiotics on IR and glycemic control, there is limited research on their impact on pancreatic β-cell function in relation to T2D (74).

An additional focus is the effect of medications on the gut microbiota. Because patients with T2D typically receive pharmacological treatment, it is challenging to distinguish which microbiota changes are directly linked to the disease. Therefore, many researchers are interested in identifying microbiota changes in individuals at the prediabetic stage, prior to medication use. Moreover, it is important to understand how pharmacotherapy for T2D influences the microbiota and thereby partly contributes to its therapeutic effectiveness in managing this metabolic disorder.

The pathogenesis of T2D involves numerous bacterial metabolites, of which *short-chain fatty acids* represent a key group.

##### 3.1.2.1 Short-chain fatty acids

In the large intestine, numerous bacterial species generate short-chain fatty acids (SCFAs) by metabolizing complex carbohydrates and proteins through various biochemical processes (64, 75). As noted above, both their absolute concentrations and relative ratios play a crucial role.

Although SCFAs are not produced by the human body itself, but rather are bacterial metabolites, they have become closely linked to molecular processes of energy generation and modulation of cellular signaling pathways in various host organs and tissues through a prolonged period of co-evolution between the microbiota and the human body. SCFAs directly enter human cells via active or passive transport and can also interact with specific receptors to initiate signal transduction processes with diverse biochemical outcomes.

Upon entering cells, SCFAs can be utilized as an energy source. For example, human colonocytes use butyrate as their primary energy substrate (76). Acetic and propionic acids are less efficient at producing ATP molecules in the tricarboxylic acid cycle and are therefore preferentially directed toward other metabolic pathways. For example, acetate is used by hepatocytes for lipid and cholesterol synthesis, while propionate undergoes hepatic conversion to glucose through gluconeogenesis (77).

SCFAs exert biological effects through their interaction with a specific group of receptors, collectively known as Free Fatty Acid Receptors (FFARs), which belong to the family of G protein-coupled receptors (GPCRs). The most studied SCFA receptors to date include Free Fatty Acid Receptor 2 (FFAR2 (GPR43)), FFAR 3 (GPR41) and GPR109A (HCA2) (78). These SCFA receptors are expressed on a variety of cell types, including intestinal epithelial cells (notably enteroendocrine I and L cells), α- and β-cells of the pancreas, and immune and neural cells.

Activation of FFAR3 by SCFAs has been demonstrated to induce the release of the hormone peptide PYY by intestinal endocrine cells. PYY enhances glucose uptake by adipose tissues and skeletal muscles, slows intestinal motility, promotes insulin secretion, and reduces appetite (79). Additionally, SCFAs binding to FFAR2 (GPR43) stimulate the secretion of glucagon-like peptide-1 (GLP-1) from intestinal L-cells (80). Consequently, this process enhances insulin secretion and decreased glucagon production by pancreatic cells. Thus, SCFAs produced by the gut microbiota modulate the secretion of hormones GLP-1 and PYY, which, through the gut–brain axis, regulate both metabolic processes and food intake (81). Moreover, SCFAs regulate blood glucose levels by affecting membrane glucose transporter proteins. Several investigations have demonstrated an inverse association between circulating acetate levels and glucose concentrations.

Furthermore, evidence indicates that SCFAs modulate the synthesis of adipokines - hormones secreted by adipose tissue - including adiponectin, leptin, and resistin, which play key roles in the regulation of metabolic processes (82, 83).

The entry of SCFAs into human cells can influence gene expression through the inhibition of histone deacetylases (HDAC), leading to hyperacetylation of certain regions of the genome. Among these metabolites, butyric acid demonstrates the strongest activity. Its cellular entry is associated with modifications in gene expression; for instance, it enhances the adiponectin-mediated activation of the AMP-activated protein kinase (AMPK) signaling pathway (84), stimulates mitochondrial biogenesis and the process of β-oxidation of fatty acids, etc. (85). Through this mechanism, butyric acid also promotes the upregulation of the *FOXP3* transcription factor expression, thereby promoting the differentiation of T cells into regulatory T cells (Tregs) (86).

SCFAs, especially butyric acid, are crucial for preserving the integrity of the intestinal epithelium, as they affect proteins responsible for the formation of tight intercellular contacts - TJP (Tight Junction Proteins). Butyric acid, through the interaction with the GPR109A receptor, enhances the expression of several TJP: claudin-3, occludin, and zonula occludens 1 (87). Increased permeability of the gut epithelium leads to the transfer of bacteria or bacterial cell components beyond the mucosal surface, thereby initiating low-grade local and systemic inflammation. This, in turn, is linked to the onset of IR and obesity.

Another important mechanism by which SCFAs influence health and pathological conditions, including T2D, involves their modulation of immune system function. Receptor for SCFAs are expressed on various types of immune cell populations, such as macrophages, neutrophils, dendritic cells, and group 3 innate lymphoid cells (ILC3s), and T cells (88, 89), which indicates that these cells are targets for the action of these bacterial metabolites. An analysis of numerous original studies indicates that the impact of SCFAs on immune mechanisms is complex, involving various pathways, but is predominantly characterized by anti-inflammatory effects (71, 73, 90, 91).

##### 3.1.2.2 Secondary bile acids

Patients with T2D demonstrate distinct profiles of secondary bile acids, a group of metabolites derived from bacterial activity, compared to healthy individuals. As is known, these secondary bile acids are produced from primary bile acids through microbial metabolism in the gut. Studies estimate that approximately 5% of bile acid conjugates undergo deconjugation and subsequent biochemical transformations mediated by intestinal microbiota enzymes. Bacterial populations in the ileum (representatives of the genera *Clostridium*, *Bifidobacterium*, *Enterococcus*, *Lactobacillus*, *Bacteroides*, *Methanobrevibacter smithii*, and *Methanosphera stadtmanae*) produce bile salt hydrolases (BSH), enzymes responsible for the deconjugation of bile acids. In addition, enzymes from other bacteria (*Clostridium* and *Eubacterium*) catalyze 7α-dehydroxylation reactions, converting primary bile acids into secondary bile acids, including deoxycholic acid (DCA), lithocholic acid (LCA), and ursodeoxycholic acid (UDCA) (52, 92). The majority of these secondary bile acids are eliminated via fecal excretion, but approximately 2% of DCA and a small amount of LCA enter the portal circulation.

Serving multiple physiological functions, bile acids, including primary and secondary forms, function as signaling molecules and can interact with specific receptors to initiate signaling cascades in responsive cells. Bile acid receptors, collectively referred to as BARs (Bile Acid Receptors). Among the most studied bile acid receptors are the nuclear receptor FXR (Farnesoid X Receptor) and the plasma membrane–expressed receptor GPBAR1 (G-Protein Bile Acid Receptor 1; Takeda G protein-coupled receptor 5, TGR5) (93). The nuclear receptor FXR is expressed in intestinal epithelial cells, hepatocytes, and the vascular endothelium of the intestine and liver. The GPBAR1 receptor is expressed on intestinal epithelial cells, muscle and neuronal cells, intestinal and liver endothelial cells, and in both white and brown adipose tissue. It is also widely present on immune cells. Both receptors can recognize primary and secondary bile acids, but their binding affinities differ, with TGR5 showing a preference for secondary bile acids produced by the gut microbiota.

FXR and TGR5 receptors signaling plays a critical role in the regulation of bile acid and lipid metabolism. Moreover, these receptors mediate the effects of bile acids on inflammation and cellular insulin responsiveness. For example, activation of FXR in pancreatic β-cells induces Forkhead box a2 (Foxa2) expression, which enhances insulin production (94). Similarly, stimulation of TGR5 on enteroendocrine L-cells promotes the release of the hormone GLP-1, thereby improving glucose homeostasis and insulin sensitivity (95).

The bile acid receptors discussed above are present on a range of immune cells, including dendritic cells, macrophages, and NK-T cells (96, 97), indicating that bile acids play a role in modulating immunoreactivity. Research indicates that bile acids, including secondary bile acids, suppress inflammation by decreasing the secretion of pro-inflammatory cytokines IL-1β, IL-6, TNF-α, IL-12, which are released by immune cells following pro-inflammatory triggers such as exposure to lipopolysaccharide (LPS) (98, 99).

It should be noted that both primary and secondary bile acids can exert toxic effects on the gut microbiota, thereby directly modulating its composition within the intestine. Conversely, bacteria of certain taxonomic groups can modulate bile acid production. For example, members of the genus *Clostridia* promote production of bile acids by suppressing FGF19 (Fibroblast Growth Factor 19), which normally bile acid production through a cascade of molecular mechanisms (100). Thus, there are complex reciprocal relationship between bile acids and the intestinal microbiota.

In individuals with T2D, increased concentrations of both primary and secondary bile acids have been observed, reflecting dysregulation of bile acid metabolism involving both the host and the gut microbiota.

##### 3.1.2.3 Tryptophan derivatives

Certain intestinal bacteria metabolize the essential amino acid tryptophan, obtained from the diet, into a variety of bioactive compounds, including indole and its derivatives, such as indole-3-aldehyde (IAld), indole-3-acetic-acid (IAA), indole-3-propionic acid (IPA), indoleacrylic acid, and indole-3-acetaldehyde (IAAld). All of them are ligands for the aryl hydrocarbon receptor (AhR) (68). This receptor is present in hepatocytes, intestinal epithelial cells, skin, endothelial cells, lungs, and different populations of immune cells. The ability to produce indole and its metabolites is found in bacteria of the genera *Escherichia*, *Proteus*, *Bacteroides*, *Clostridium*, *Peptostreptococcus, Lactobacillus, Enterococcus, Eubacterium, Anaerostipes, Bifidobacterium* (101). The abundance of these bacterial species in the gut microbiota, together with the tryptophan content of the diet determine the levels of corresponding metabolites produced in the gut and circulating in the bloodstream (102–104). The effects of AhR activation are context-dependent. For example, In intestinal epithelial cells, AhR activation promotes cellular differentiation and increases the expression of tight junction proteins, including ZO-1, Occludin, Claudin-1, thereby enhancing epithelial barrier function (105). AhR activation in immune cells (in Th17 and Th22 cells) stimulates the secretion of IL-22, supporting mucosal integrity (106). AhR signaling participates in M2-type macrophage activation, resulting in IL-10 production (107) and promotes the differentiation of tolerogenic dendritic cells (108).

Investigations of the association between indole derivatives and T2D have shown the following: higher plasma concentrations of ІРА are linked to enhanced insulin secretion and sensitivity, reduced chronic low-grade inflammation, accompanied by a decreased risk of T2D onset (109). Another study demonstrated that serum IPA levels were markedly reduced in patients with DKD and showed significant correlations with urine albumin-to-creatinine ratio (UACR), estimated glomerular filtration rate (eGFR), fasting blood glucose and HbA1c (110). In a high-fat diet (HFD) mouse model, intraperitoneal administration of IAA improved liver function, reduced fasting glucose levels, and normalized the lipid profile (111).

#### 3.1.3 Immunoreactivity and microbiota

Gut microbiota-stimulated immune mechanisms contribute significantly to the development of T2D. Studies show that T2D develops against the background of chronic low-grade systemic inflammation. Factors that sustain this inflammation include bacterial components (РАМРs or MAMPs) that enter the bloodstream from the intestine, which becomes “leaky” (leaky gut) and act systemically. Research has demonstrated that the blood serum of individuals diagnosed with T2D presents with markedly elevated levels of one of the most extensively studied MAMPs, lipopolysaccharide (LPS). MAMPs interact with receptors, such as Toll-like receptors (TLRs). These receptors are often found on the cells of the epithelium and immune system. The stimulation of these receptors results in the secretion of pro-inflammatory cytokines (IL-1, IL-6, TNF-α) by cells, exerting a systemic effect. Impairment of normal functioning of pancreatic β-cells and the development of IR in various tissues have been associated with these immune processes. Furthermore, systemic inflammation affects endothelial function. Prolonged exposure to pro-inflammatory factors activates endothelial cells, causing them to produce pro-inflammatory cytokines and express more adhesion molecules, which promotes the attachment of activated leukocytes and platelets. These processes play a role in the development of atherosclerotic lesions (112–114).

As we can see, the described immune mechanisms involved in the pathogenesis of T2D are interdependent with the gut microbiota. The increased abundance of opportunistic bacteria with pro-inflammatory properties in the microbiota leads to the translocation of bacteria and their components into the bloodstream, thereby exacerbating the systemic inflammatory response (53, 115).

#### 3.1.4 Approaches to modulating the gut microbial composition in individuals with T2D

So, the gut microbiota plays a central role in the pathogenesis of T2D. What factors regulate the structure and metabolic activity of intestinal microbiota? The key factors currently recognized for modulating the gut microbiota include diet, physical activity, probiotic use, and fecal microbiota transplantation (FMT).

In our view, the impact of diet on the microbiota is the most important factor (116). The strongest evidence of how a human’s diet affects their microbiota can be observed during the transition from breastfeeding to an adult diet (117, 118). Research shows that changes in diet, or even the regular consumption of certain foods, can modify gut microbiota composition and function, leading to improvements in both clinical outcomes and biochemical markers in patients with T2D (119, 120).

Different regions of the world have their own national cuisines and eating habits. The effect that different diets have on the gut microbiota is currently being studied, as is the subsequent effect on human health. For a long time, the Mediterranean diet (MeD) has been one of the most researched diets in regard to its beneficial effect on the microbiota and overall human health (121, 122). There are many original studies and analytical summaries on the beneficial role of the MeD in reducing the risk of type 2 diabetes and improving the clinical picture and laboratory test results in already developed disease. A large prospective study conducted in Spain showed that adherence to the Mediterranean diet can prevent the onset of T2D (123). Moreover, a comprehensive meta-analysis demonstrated that following the MeD in individuals with T2D resulted in reductions in fasting plasma glucose and insulin, HbA1c, BMI and body weight. In addition, triglycerides and total cholesterol concentrations in plasma decreased, while high-density lipoprotein concentrations increased. Furthermore, patients demonstrated a decrease in blood pressure (124). This positive effect of MeD is linked to its effects on the gut microbiota (125). The low intake of animal proteins and the high consumption of dietary fibers characteristic of this dietary pattern lead to modifications in the microbiota composition, increasing the number of bacteria such as *Roseburia* spp, *Akkermansia muciniphila* and *Faecalibacterium prausnitzii*. These bacteria ferment dietary fibers and produce SCFAs (126, 127) with various other health-promoting metabolites.

Physical activity is an important factor that influence the composition of the human gut microbiota positively. Research has demonstrated that a lack of physical activity is linked to decreased gut microbiota diversity and an elevated Bacillota/Bacteroidota ratio, which is frequently observed in metabolic disorders (128). Moderate-intensity physical activity has been shown to increase the levels of gut bacteria from the genera *Faecalibacterium*, *Veillonella*, *Lachnospira*, and *Bifidobacterium*, which are linked to improved metabolic profiles and anti-inflammatory activity (129, 130). Multiple studies indicate that moderate physical activity helps normalize gut microbiota balance, potentially improving metabolic profiles and providing an additional beneficial effect in the treatment of T2D.

Specialists in the treatment of T2D have also given positive reviews on the correcting of the microbiota using probiotic preparations. Numerous meta-analyses have examined the effect of probiotic supplements on the effectiveness of treating T2D. They have shown that the use of probiotics improves metabolic parameters such as fasting glucose, insulin, and HbA1c in patients with T2D (131–133). Most studies traditionally used currently available probiotics based on bacteria of the *Bifidobacterium* and *Lactobacillus* genera. Another study investigated the impact of a probiotic containing 12 bacterial strains (*Bifidobacterium, Streptococcus* and *Lactobacillus*) adjunctive therapy for patients with T2D and hyperammonaemia. After one month of supplementation, patients showed reduced peripheral blood levels of fasting glucose and ammonia. In addition, the patients experienced changes in their gut microbiota, with a decrease in the number of bacteria species with pro-inflammatory properties.

In light of the current success of probiotics, there is now talk of developing the next-generation, which will be based on a wide variety of bacteria with beneficial properties, such as *Akkermansia muciniphila*, *Faecalibacterium prausnitzii*, *Anaerobutyricum soehngenii*, *Roseburia hominis*, and *Cristensenella minuta* (134, 135), which will have a positive effect on human health in both normal conditions and various pathological processes.

Modifications to the microbiota through the use of probiotics or dietary interventions occur relatively slowly, typically manifesting after 1–3 months of exposure. A faster way to modulate the gut microbiota is through fecal microbiota transplantation (FMT). This approach is currently being actively developed and has already proven effective in treating pathological conditions, including recurrent *Clostridioides difficile* infection (CDI), inflammatory bowel disease (IBD), irritable bowel syndrome (IBS), neurodegenerative disorders and autoimmune diseases (136–139). However, the number of studies on the application of FMT to patients with T2D is still limited, preventing definitive conclusions regarding the efficacy of this procedure. Some authors have reported positive effects of FMT (140), whereas others observed no beneficial outcomes in patient groups with high IR (141). Therefore, research in this area is ongoing.

The available evidence clearly indicates the critical role of the gut microbiota in the pathogenesis of T2D (142).

#### 3.1.5 Influence of regional dietary patterns on gut microbiota and T2D

Regional dietary patterns have a significant influence on gut microbiota composition and, consequently, the pathophysiology of type 2 diabetes (T2D). Diets vary widely across geographic regions due to cultural, economic, and environmental factors, which impact the production of microbial metabolites, such as short-chain fatty acids (SCFAs), and their role in maintaining metabolic health. For instance, high-corn diets, prevalent in Latin America, are rich in dietary fiber, which promotes the growth of SCFA-producing bacteria such as *Faecalibacterium* and *Roseburia*. These bacteria are associated with enhanced insulin sensitivity and reduced inflammation, potentially mitigating the risk of T2D (125). In contrast, high-red-meat diets, common in Australia, may increase the abundance of pro-inflammatory bacteria and elevate levels of metabolites, such as trimethylamine-N-oxide (TMAO), which is linked to insulin resistance and an increased risk of T2D (62). These regional differences highlight the need for further research to elucidate how specific dietary patterns shape microbiota-T2D interactions, particularly in underrepresented populations. Such studies could inform the development of tailored dietary interventions to optimize gut microbial profiles and improve T2D management globally.

### 3.2 Current applications of artificial intelligence and machine learning in the diagnosis and management of diabetes mellitus

#### 3.2.1 Machine learning models for early diagnosis, risk assessment, and complication prediction

Artificial intelligence, particularly machine learning methods, plays a central role in the early detection of diabetes mellitus, assessment of disease risk, and prediction of complications such as diabetic retinopathy, nephropathy, and cardiovascular disorders. To analyze large datasets, including electronic health records (EHRs), genetic profiles, and lifestyle indicators, various models are employed, including logistic regression, decision trees, random forest, support vector machines (SVM), and deep neural networks.

Nomura et al. demonstrated that machine learning models achieved area under the receiver operating characteristic curve (AUC) values ranging from 0.71 to 0.80 when predicting the development of T2D over a five-year period. These predictions were based on clinical parameters, including HbA1c levels, body mass index (BMI), and genetic predisposition (143). For instance, a logistic regression model proposed by Choi et al. showed an AUC of 0.78 for predicting T2D in hospitalized patients using demographic and laboratory data (144). Ravaut et al. reported an AUC of 0.80 when analyzing administrative health data for T2D risk assessment (145). Similarly, Yun et al. developed a deep learning–based system for risk stratification of T2D using retinal images, enabling the detection of early pathological changes with accuracy comparable to expert assessments (146).

For patients with T2D, the risk of complications is a significant concern, and substantial attention has been focused on the use of AI in this context. For instance, Betzler et al. utilized deep learning techniques to predict diabetic nephropathy using retinal photographs, achieving an area under the curve (AUC) of 0.85 (147). Machine learning (ML) models are also applied to assess cardiovascular risk in individuals with diabetes mellitus. In the ORFAN STUDY Chan et al. demonstrated that AI can predict cardiovascular alterations in patients without obstructive coronary artery disease using computed tomography (CT) data combined with clinical parameters (148). These models integrate imaging data with biomarkers, such as glucose levels and lipid profiles, to generate comprehensive risk profiles.

Khalid et al. highlights the effectiveness of Gradient Boosting and XGBoost algorithms, which can predict T2D with an AUC of up to 0.87 based on a combination of genetic data and EHRs (149). Furthermore, deep learning methods have demonstrated the ability to detect subclinical manifestations of diabetic complications, such as microvascular alterations, thereby enhances the accuracy of early diagnosis. These ML algorithms can be further enhanced by integrating gut microbiota data, such as metabolomic profiles (e.g., short-chain fatty acids) from Section 3.1, to identify novel biomarkers, like cysteine or phenyllactate, that correlate with insulin resistance (150).

Several ML algorithms have been employed to enhance T2D management. Random Forest and XGBoost, tree-based ensemble methods, excel in ranking feature importance using techniques such as Shapley Additive Explanations (SHAP), which helps identify key biomarkers, including HbA1c, folate, and metabolites like cysteine and aspartate, in metabolomic studies (145). These algorithms are robust for high-dimensional datasets and have shown high accuracy in predicting T2D risk and complications, such as distal symmetric polyneuropathy (144). Support Vector Machines (SVMs) are effective for classifying molecular biomarkers, such as long non-coding RNAs (lncRNAs), in high-dimensional genetic data, and for diagnosing T2D using clinical and imaging data (144). Deep Neural Networks (DNNs) are particularly suited for multimodal data integration, capturing complex non-linear patterns in time-series data (e.g., continuous glucose monitoring) and retinal photographs to predict glycemic control and diabetic retinopathy progression (151). Ensemble methods like Voting and Stacking combine the strengths of multiple models to improve generalization and biomarker ranking across metabolomics and genetics. Logistic Regression, while simpler, serves as a baseline for interpretable risk assessment and is often used in ensemble approaches (145).

Recent studies have demonstrated the efficacy of these algorithms in the management of T2D. For instance, a machine learning model using administrative health data achieved an AUC of 0.957 for predicting T2D onset within five years (145). Another study utilizing DNNs on retinal photographs reported an AUC of 0.934 for detecting diabetic kidney disease, highlighting the potential of AI in early detection of complications (144). Additionally, XGBoost models applied to the NHANES dataset identified novel metabolomic biomarkers, such as phenyllactate, with an AUC of 0.86 for insulin resistance prediction (120, 152). These advancements underscore the synergy between AI-driven diagnostics and gut microbiota research, enabling the identification of novel therapeutic targets and personalized interventions.

A comprehensive comparison of these ML algorithms, including their applications in T2D biomarker identification and diagnosis, their advantages and limitations, and performance metrics, is provided in **Table 2**.

**Table 2:** Machine learning algorithms for biomarker identification and diagnosis of type 2 diabetes.

Algorithm	Category	Application in identifying T2D biomarkers	Application in disease diagnosis	Advantages	Limitations	Performance examples
Random Forest	Tree-based Ensemble	Used for feature importance ranking (e.g., Shapley Additive Explanations) to identify biomarkers such as blood glucose levels, HbA1c, folate, lncRNA in metabolomics and genetics. Effective for high-dimensional data with nutritional markers.	Predicting T2D risk, classifying stages (e.g., prediabetes/T2D), detecting complications like distal symmetric polyneuropathy. Used for early diagnosis with electronic medical records and imaging.	High resistance to overfitting, handles imbalanced data, interpretable through feature importance; performs well with tabular data.	Less effective on very large datasets without tuning; “black box” without Shapley Additive Explanations.	AUC 0.99 (symptoms), 0.835 (long-term prognosis); accuracy 0.975 with feature interactions.
XGBoost (Gradient Boosting)	Gradient Boosting	Identification of key biomarkers (e.g., cysteine, aspartate, phenyllactate) via Shapley Additive Explanations in metabolomics; ranking risk factors like BMI, blood glucose levels.	Predicting insulin resistance, complications (hypoglycemia, distal symmetric polyneuropathy), long-term risk, and precise diagnosis with electronic medical records.	High accuracy, handles missing data, regularization against overfitting; scales to large datasets.	Prone to overfitting without regularization; requires hyperparameter tuning.	AUC 0.957 (NHANES), 0.86 (insulin resistance); accuracy 71-73% for the following year.
Support Vector Machine	Kernel-based Classifier	Detection of molecular biomarkers (e.g., lncRNA) in high-dimensional genetic data; classification based on metabolites.	Diagnosis with clinical and imaging data; ensembles for detecting retinopathy and progression.	Effective in high-dimensional spaces, resistant to overfitting due to margins; suitable for non-linear boundaries with kernels.	Slow training on large datasets; requires normalization; less interpretable.	AUC 0.95 (lncRNA), 0.928 (clinical data); sensitivity 95%, specificity 86%.
Deep Neural Networks	Neural Networks	Analysis of multimodal data (electronic medical records + genetics/imaging) to identify novel biomarkers (e.g., metabolites, genetic risks).	Predicting glycemic control, progression from prediabetes; processing time series and images for diagnosis.	Captures complex non-linear patterns; scales to large datasets; supports multimodal integration.	Requires large datasets; computationally intensive; prone to overfitting.	AUC 0.934 (fused data), accuracy 92-94% (pipelines); low RMSE for glucose prediction.
Voting, Stacking	Combined Models	Combining for biomarker ranking from various sources (metabolomics + genetics); improves interpretability with Shapley Additive Explanations.	Multiclass classification (healthy/prediabetes/T2D); long-term risk prediction with electronic medical records.	Leverages strengths of base models; improves generalization and balances metrics.	Increased complexity and computation time; depends on base model quality.	Accuracy 99.3%, F1 0.993 (DT/SVM/XGBoost); AUC 0.884 (weighted voting).
Logistic Regression	Linear Model	Baseline for detecting simple biomarkers (e.g., nutritional markers); used in ensembles for interpretable coefficients.	Baseline risk diagnosis; ensembles for current T2D status.	Simple, interpretable (odds ratios); effective for small datasets.	Assumes linear relationships; performs poorly on non-linear data; sensitive to multicollinearity.	AUC 0.746-0.884 (in ensembles); baseline for comparisons.

#### 3.2.2 Analysis of continuous glucose monitoring data using machine learning methods

Continuous glucose monitoring (CGM) systems generate large volumes of data that AI can use to predict glycemic events such as hypoglycemia and hyperglycemia. Deep learning algorithms, particularly convolutional neural networks (CNNs) and recurrent neural networks (RNNs), effectively analyze CGM time-series data to identify patterns and predict glucose levels.

A meta-analysis by Kodama et al. showed that models like RNNs and long short-term memory (LSTM) networks can achieve a mean absolute error of 10–15 mg/dL in predicting glucose levels 30 minutes before an event (153). Jaloli and Cescon developed a hybrid CNN-LSTM model that can predict long-term glucose levels in people with type 1 diabetes mellitus (T1D) with up to 90% accuracy (154). The Guardian Connect System by Medtronic, approved by the U.S. Food and Drug Administration (FDA) in 2018, uses predictive algorithms to provide alerts up to one hour before a potential hypoglycemic event, with 98.5% accuracy at 30 minutes before onset (11).

The main challenges in glucose prediction are delays associated with food and insulin absorption, as well as CGM errors, which account for approximately 9% of the mean absolute relative difference for the most accurate sensors. To overcome these limitations, multimodal models are employed that integrate CGM data with information on physical activity, nutrition, and sleep obtained from individual wearable devices. For example, Guan et al. demonstrated that integrating CGM data with fitness tracker information improves glucose prediction accuracy by 15% (155). Closed-Loop Insulin Delivery Systems, such as the artificial pancreas, implement these models to automatically regulate insulin delivery, thereby reducing glycemic variability by 25% in patients with T1D.

#### 3.2.3 Integration of microbiome data into diagnostic algorithms

##### 3.2.3.1 The development of diagnostic algorithms based on the microbiome is a growing area of research

The gut microbiome plays a crucial role in metabolic processes and the development of T2D, particularly through its influence on insulin resistance and inflammatory responses. AI algorithms are applied to analyze metagenomic data, which reflect the composition of the microbiota, as well as metabolomic profiles, including SCFAs, to create diagnostic models.

Lagou et al. demonstrated that multimodal models integrating metagenomic data with EHRs achieved an AUC of 0.82 for predicting the risk of T2D (150). Liu et al. developed a gradient boosting–based algorithm that analyzes the composition of the microbiota and its metabolites to identify metabolic phenotypes of T2D. Notably, elevated butyrate levels are associated with reduced IR, making it a potential biomarker (156). Karlsson et al. found that a decrease in bacteria of the genus *Roseburia* correlates with an increased risk of developing T2D, which may inform the development of bioactive therapeutic strategies (58).

AI algorithms also enable the analysis of complex interactions between the microbiome and metabolic pathways. For example, Qin et al. developed a catalog of gut microbiome genes used to identify biomarkers of T2D, including genes associated with the synthesis of SCFAs (157). Algorithms such as SVM and Random Forest have been shown to effectively classify microbial profiles for predicting metabolic disorders.

**Figure 2:**
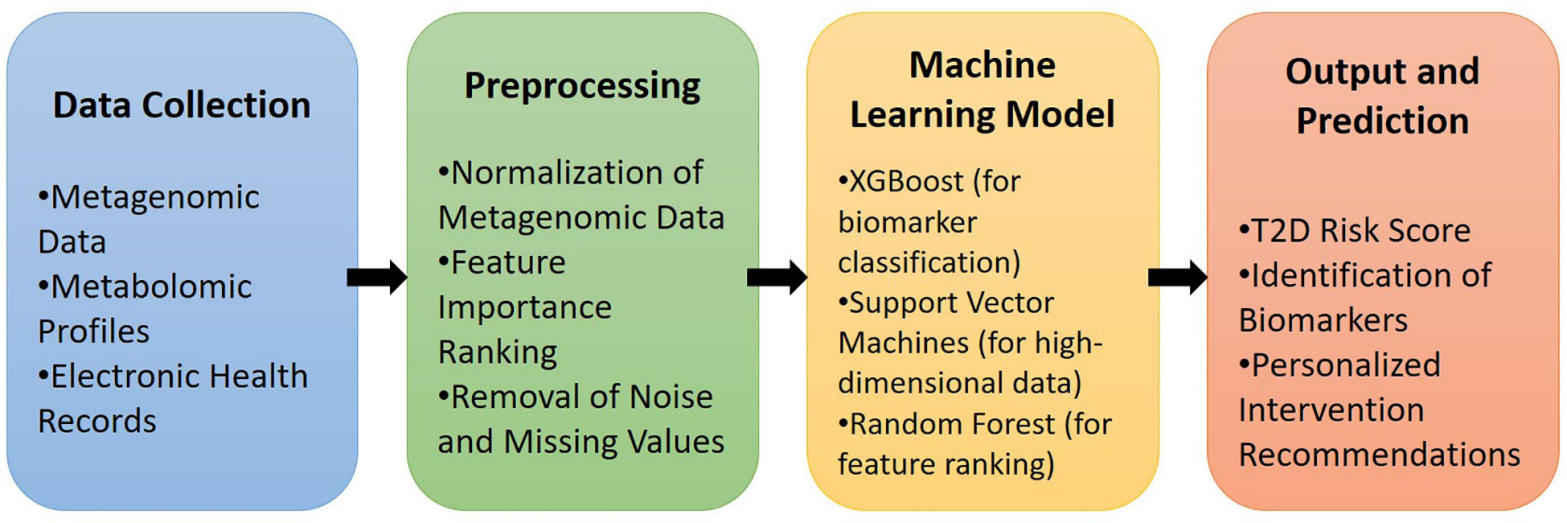
Pipeline for machine learning integration with gut microbiome data in T2D diagnosis.

**Figure 2** illustrates the workflow for integrating gut microbiome data (metagenomic and metabolomic profiles) with machine learning algorithms to facilitate the early diagnosis and risk prediction of T2D, highlighting key stages from data collection to personalized intervention recommendations.

##### 3.2.3.2 Patient stratification based on microbiota composition and bioactive profiles

AI can categorize patients with diabetes based on their microbiota composition, enabling personalized treatment strategies. For example, the DayTwo platform employs algorithms to analyze metagenomic data and predict glycemic responses based on dietary intake. A study by Zeevi et al. demonstrated that such algorithms can predict individual glycemic responses with up to 70% accuracy using microbiota composition and dietary data (158). This approach facilitates the development of personalized dietary recommendations that reduce glycemic spikes.

Stratification also facilitates the identification of patient subgroups with varying risk of complications. A study by González-Rivas et al. demonstrated that AI platforms can classify patients according to their level of insulin resistance based on microbiota composition, enabling tailored probiotic therapy. Specifically, probiotic strains such as *Lactobacillus* and *Bifidobacterium*, increase butyrate levels, which are associated with reduced inflammatory markers and improved glycemic control. These platforms employ clustering methods (e.g., k-means) and causal inference analyses to tailor therapy to metabolic profiles, thereby reducing the risk of complications, such as diabetic nephropathy, by 15–20% (159).

##### 3.2.3.3 Platforms for analyzing metagenomic and metabolomic data

Modern platforms, such as IBM Watson Health and Google DeepMind, are applied to process large-scale metagenomic and metabolomic datasets. For instance, the DayTwo platform integrates microbiome data with EHRs to generate personalized dietary recommendations (158).

Processing multimodal data is a complex task that requires algorithmic advancements to ensure interpretability and scalability. For example, deep learning methods, such as autoencoders, enable dimensionality reduction while preserving key biomarkers, but they demand substantial computational resources (160).

#### 3.2.4 Digital pathology and biomarker identification

##### 3.2.4.1 Identification of microbial metabolites associated with disease progression

AI algorithms play a crucial role in discovering new biomarkers, including microbial metabolites that influence the progression of T2D. Deep learning enables the analysis of metabolomic profiles to identify metabolites, such as butyrate and propionate, which correlate with IR. A study by Santhanam et al. demonstrated that these methods can assess body composition, including visceral fat, through CT and MRI image analysis, which is an important risk factor for the development of T2D (161). Specifically, the algorithms revealed that elevated levels of visceral fat are associated with a 30% higher risk of developing T2D.

Karlsson et al. highlighted that metagenomic data can be used to identify biomarkers of T2D, including metabolites that influence inflammatory processes (58). Algorithms such as k-means clustering enable the categorization of metabolic profiles to identify new therapeutic targets.

##### 3.2.4.2 Image analysis and systems biology in synergy with microbiome data

Digital pathology using AI algorithms is widely applied for diagnosing diabetic complications, particularly diabetic retinopathy. For example, Rice et al. developed a deep learning system for automated detection of retinopathy from fundus images, achieving 97% sensitivity and 95% specificity (162). Dai et al. developed a deep learning system to predict the progression of diabetic retinopathy, achieving an AUC of 0.90 (151). These systems integrate retinal images with clinical data to create complex diagnostic models.

Such methods also allow the establishment of associations between microbial metabolites and pathological tissue changes. For example, a study by Liu et al. demonstrated that 21 identified microbial genera are important biomarkers for T1D. Their AUC values were 0,962 and 0.745 on discovery set and validation set. Functional analysis indicated that 10 microbial genera were significantly positively correlated with D-arginine and D-ornithine metabolism, transcriptional spliceosome activity, steroid hormone biosynthesis and glycosaminoglycan degradation (156). These findings support the prediction of disease progression and the development of personalized therapeutic strategies.

#### 3.2.5 Convergence toward personalized therapeutic platforms

##### 3.2.5.1 Therapeutic platforms based on gut-derived bioactive compounds

Bioactive metabolites synthesized by the gut microbiota, particularly SCFAs, such as butyrate and propionate, play a crucial role in regulating metabolic and inflammatory processes associated with T2D. As noted above, these compounds influence IR, glycemic control, and levels of inflammatory markers, such as C-reactive protein. AI enables the development of therapeutic platforms that analyze microbiota composition and its metabolites to design individualized strategies, including the use of probiotics, postbiotics, and dietary recommendations.

A study by Zeevi et al. demonstrated that these algorithms facilitate personalized dietary recommendations, which help reduce postprandial glycemic spikes in patients with T2D, ultimately lowering the risk of complications such as diabetic retinopathy and cardiovascular disease. They employed the DayTwo platform, which uses AI algorithms, including gradient boosting, to analyze gut microbiome metagenomic data and predict diet-based glycemic responses, achieving up to 70% accuracy in forecasting individual glycemic responses (158).

###### 3.2.5.1.1 Integration of omics data with lifestyle information

The integration of multimodal omics data, including microbiome, metabolomic, and genomic profiles, with lifestyle information (diet, physical activity, and sleep) forms the foundation for the development of personalized therapeutic platforms. A study by Lagou et al. further demonstrated that multimodal models combining UK Biobank genomic data, gut microbiome metagenomic profiles, and EHRs achieved an AUC of 0.85 for predicting the risk of developing T2D (150). These models employ gradient boosting algorithms, such as XGBoost, and deep learning methods to identify biomarkers, including SCFAs, that correlate with metabolic phenotypes.

For example, elevated levels of butyrate are associated with reduced IR, whereas a decrease in the genus *Faecalibacterium* is associated with an increased risk of developing T2D (150). A study by Kannenberg et al. demonstrated that an AI platform based on digital twins reduced HbA1c by 1.2% over 12 months in patients with T2D by adapting dietary recommendations based on microbiome and metabolome data (163). Integration of data from individual monitoring devices, such as fitness trackers, enables the creation of comprehensive risk profiles.

Qin et al. developed a catalog of gut microbiome genes, which is used to identify biomarkers of T2D, including genes associated with SCFAs synthesis (157). Algorithms such as SVM and Random Forest enable the classification of metabolic profiles with up to 80% accuracy for predicting the risk of developing T2D.

Integration of lifestyle data, such as dietary caloric intake and physical activity, improves predictive accuracy by 10–15%. For example, the Twin Health platform uses multimodal data to generate personalized nutrition plans that reduce glycemic peaks in patients with T2D.

###### 3.2.5.1.2 Adaptive algorithms for real-time monitoring and therapy correction

Adaptive algorithms, such as reinforcement learning (RL) and RNNs, enable real-time patient monitoring and dynamic therapy correction. A study by Guan et al. showed that RL algorithms optimize insulin dosing in T2D, reducing the risk of hypoglycemia by 30% compared to traditional methods (155). These algorithms integrate data from CGM, physical activity, and EHRs to develop dynamic therapeutic strategies.

The Advisor Pro system by DreaMed Diabetes, approved by the FDA in 2018, uses algorithms to analyze CGM data and provide real-time insulin dosing recommendations, improving glycemic control in patients with T1D (164). A meta-analysis by Kodama et al. established that such models reduce the incidence of hypoglycemic events by 20% compared to traditional methods. LSTM-based algorithms achieve a mean absolute error of 10–15 mg/dL when predicting glucose levels 30 minutes in advance, enabling timely therapy corrections (153).

The main challenges include delays in CGM data (5–10 minutes) and the need to standardize information from multiple sources, such as wearable devices and EHRs (155). To overcome these challenges, multimodal models are employed, integrating CGM with information on diet, physical activity, and sleep.

These models facilitate the development of closed-loop systems that automatically regulate microdoses of insulin, thereby reducing glycemic variability and improving overall metabolic control.

##### 3.2.5.2 Potential of remote care, digital twins, and closed-loop systems

###### 3.2.5.2.1 Remote care

Remote care platforms, such as MyWay Digital Health, enable patient monitoring through telemedicine systems by analyzing CGM data, EHRs, and information from individual monitoring devices. A study by Mackenzie et al. demonstrated that such platforms increase therapy adherence by 20% through personalized recommendations and remote consultations (165). Telemedicine reduces barriers to healthcare access, particularly in resource-limited settings, such as rural areas, remote regions, or low-income countries.

The BlueStar platform by WellDoc employs machine learning methods to analyze CGM data and provide lifestyle recommendations, reducing HbA1c by 0.8% over six months in patients with T2D (160). A study by Xu et al. demonstrated that telemedicine platforms increase healthcare accessibility by 25% compared to traditional approaches (166). These platforms integrate data from individual monitoring devices, such as Fitbit or Apple Watch, to generate comprehensive health profiles.

###### 3.2.5.2.2 Digital twins

Digital twins are virtual patient models that integrate CGM, microbiome, genomic, and lifestyle data to simulate metabolic profiles. A study by Kannenberg et al. demonstrated that digital twins can predict diabetes-related complications with up to 85% accuracy and optimize therapy through personalized dietary and pharmacological interventions. For instance, the Twin Health platform employs digital twins to design individualized nutrition plans, reducing postprandial glycemic peaks in patients with T2D by 20% (163).

Digital twins also enable the modeling of long-term therapeutic outcomes. González-Rivas et al. showed that digital twins integrating microbiome and genomic data can predict the risk of diabetic nephropathy with 80% accuracy (159). These models apply deep learning algorithms to analyze multimodal data and construct individualized risk profiles.

###### 3.2.5.2.3 Closed-loop systems

Closed-loop systems, such as the artificial pancreas, use algorithms to automatically regulate insulin delivery based on CGM data. A study by Sheng et al. reported that such systems reduce glycemic variability by 25% in patients with T1D (167). RL algorithms adapt to changes in physiological condition, providing continuous therapy adjustments. For example, the Control-IQ system by Tandem Diabetes Care, approved by the FDA in 2019, reduces the incidence of hypoglycemia by 30% (168).

Unsworth et al. demonstrated that closed-loop systems integrating CGM and individual monitoring devices improve glycemic control in children with T1D, lowering HbA1c by 0.5% over six months (164).

## 4 Discussion

### 4.1 Limitations, challenges, and ethical issues of AI in T2D management

Despite growing enthusiasm for the use of gut-derived bioactive compounds and AI in clinical practice, particularly in the monitoring of patients with T2D, several challenges limit their widespread implementation. We aimed to systematize and outline the key limitations that currently constrain the scalability of AI in diabetes management.

#### 4.1.1 The heterogeneity of the gut microbiome

remains one of the most critical barriers, complicating the standardization of bioactive markers and their application in predictive models. High interindividual variability in the gut microbiota composition of patients with T2D (169–171) prevents the direct extrapolation of findings from one population to another. It is crucial to highlight that a significant area for future research involves examining data from regions that were underrepresented in our review, particularly Latin America and Australia. Additionally, it is important to investigate how various dietary patterns—such as high-corn diets in Latin America and high-red-meat diets in Australia—affect the production of SCFAs and the composition of gut microbiota, as well as their influence on T2D. This complicates the identification of universal diagnostic and therapeutic targets.

#### 4.1.2 The technical limitations

There are problems with data unification and quality. Incomplete, unrepresentative or unstructured datasets, in particular, contribute to model bias and limited generalizability. The implementation of closed-loop systems and machine learning algorithms is constrained by the high cost of these systems, the need for data standardization to ensure compatibility across different devices, and subsequent unification and scalability. Currently, most machine learning algorithms are trained on limited demographic or regional datasets, which can lead to reduced effectiveness for smaller, underrepresented populations or patients from different parts of the world (172). Pagano et al. reviewed current machine learning models and noted that most studies focus on binary classification tasks, whereas the multidimensional clinical scenarios (173), which are typical for T2D, remain underdeveloped, limiting the scalability of these technologies for global application.

#### 4.1.3 The opacity of decision-making processes

Deep neural networks, frequently used to analyze CGM, microbiome or metagenomic data, have a complex structure and low transparency in decision-making. Most AI models are “black boxes”, meaning they produce results without explanation. This complicates their use in clinical practice, where explainable logic is required. For example, even high-accuracy models, such as CNN-LSTM that predict glucose levels with 90% accuracy, raise questions about the underlying mechanisms of their decisions (154).

#### 4.1.4 Interdisciplinary barriers

Computational models used to study the dynamic behavior of complex systems require a comprehensive combination of biological, clinical, and computer data (149). However, this requires interoperable tools, shared repositories and agreed modelling standards (174). These factors significantly complicate collaboration between specialists in bioinformatics, microbiology, clinical nutrition and endocrinology.

#### 4.1.5 Ethical aspects

Ethical aspects involve a wide range of diverse moral and legal responsibilities. Systems that process personal medical information, including genomics, microbiome or physical activity data, always carry risks of data breaches or unauthorized use. Integrating AI into healthcare raises new ethical challenges, including issues of informed consent, protection of confidential information and algorithmic transparency. Modern AI systems analyze multimodal data: from daily lifestyle to microbiota, metabolomics, genomics, etc., which requires the development of new legal mechanisms to protect privacy. Current regulatory approaches, such as GDPR in the EU and HIPAA in the USA, are not fully adapted to the specific characteristics of AI models that process multimodal data streams (175).

The use of autonomous or semi-autonomous systems, such as artificial pancreases and voice-activated digital assistants, raises questions about responsibility for clinical decisions. Even with high prediction accuracy (up to 92% for glucose prediction), AI systems are unable to fully consider the psycho-emotional, behavioral, and social factors that are critical for the long-term management of chronic diseases (167). Even the most advanced models can produce inaccurate predictions, especially if the data is incomplete or of poor quality. This can lead to incorrect insulin dosing or missed complications. The ethical dilemma of who is responsible for decisions made by AI remains unresolved. Is it the doctor, the patient, or the developer? This question becomes particularly acute in cases of complications or harm.

Currently, adaptive management strategies and international collaboration are necessary to address the global challenges of AI development, ensuring a balance between innovation and the protection of individual rights and societal values (176).

#### 4.1.6 Inequity in access to technology

High-tech solutions that require continuous internet access, modern smartphones or sensor devices (e.g., CGM) may be inaccessible to low-income patients. The rapid pace of AI development may also pose barriers for older adults. This threatens to exacerbate healthcare disparities among different socioeconomic groups (177).

#### 4.1.7 Overreliance on technology

Current solutions, such as closed-loop algorithms, mobile monitoring apps, voice assistants and digital coaches, have already demonstrated clinical benefits, including improvements in HbA1c levels, reductions in BMI and enhanced self-management. Platforms such as DiabetesCoach (178) or Healthy at Home (179), have been implemented as part of digital coaching programs with measurable behavioral effects. However, the increasing integration of digital technologies in T2D management raises discussions regarding not only their effectiveness, but also the potential risks of overreliance.

Patients may lose self-management skills if they rely solely on closed-loop algorithms. In the absence of device access or in the event of technical failure, such dependence poses risks of clinical complications. At the same time, emotional dependence may develop: digital assistants, especially those that adapt to user behavior, can create a sense of social presence. These quasi-social bonds, formed in the absence of human interaction, can contribute to digital alienation and impair social skills. Patients who interact daily with algorithms may gradually lose the ability for empathy and social flexibility (180, 181). This is especially dangerous in cases of system failure - patients who rely solely on technology may be unprepared to act independently. Idealizing an AI partner can also lead to unrealistic expectations of real people, causing frustration in interpersonal relationships. This is not just a side effect of digitalization; it is potentially a new form of patient vulnerability.

### 4.2 Conclusions and future perspectives

Bioactive compounds originating from the gut microbiota, such as short-chain fatty acids, secondary bile acids, and tryptophan metabolites, present a new therapeutic opportunity in metabolic health. Their ability to modulate hormone secretion, epithelial barrier permeability, immune response regulation, and metabolic homeostasis positions these molecules as promising targets for interventions in T2D. At the same time, the rapid advancement of AI technologies provides unprecedented opportunities to process large datasets and decipher complex bioinformatic interactions among the genome, metabolome, microbiome, and behavioral determinants. This enables the identification of individual response patterns to diet or therapy (182), providing a basis for personalized, adaptive therapeutic models, that account not only for metabolic markers but also for psychological status, social determinants, and healthcare access (183).

Integrating gut microbiota data with the analytical capabilities of AI models (digital twin systems, deep learning models, and closed-loop systems) has already demonstrated positive outcomes in optimizing glycemic control, predicting complications, and individualizing therapy. Platforms such as DayTwo, Twin Health, mySugr and MiniMed 780G confirm the practical feasibility of integrating AI with microbiome-based approaches in next-generation endocrinology. A comparison of these AI platforms is presented in **Table 3**.

**Table 3:** Comparison of AI platforms for T2D management, highlighting their functions, evidence, and limitations.

Platform	Function	Limitations	Evidence base
DayTwo	Personalized dietary recommendations based on gut microbiome analysis	Limited to dietary interventions; requires microbiome profiling	(158)
Twin Health	Holistic digital twin for metabolic health management, including glycemic control	High cost; requires continuous data input	(161)
mySugr	Glucose monitoring and behavioral coaching via app	Focuses on lifestyle, less on microbiome	(163)
MiniMed 780G	Automated insulin delivery with closed-loop system	Hardware-dependent; not microbiome-integrated	(164)

**Figure 3:**
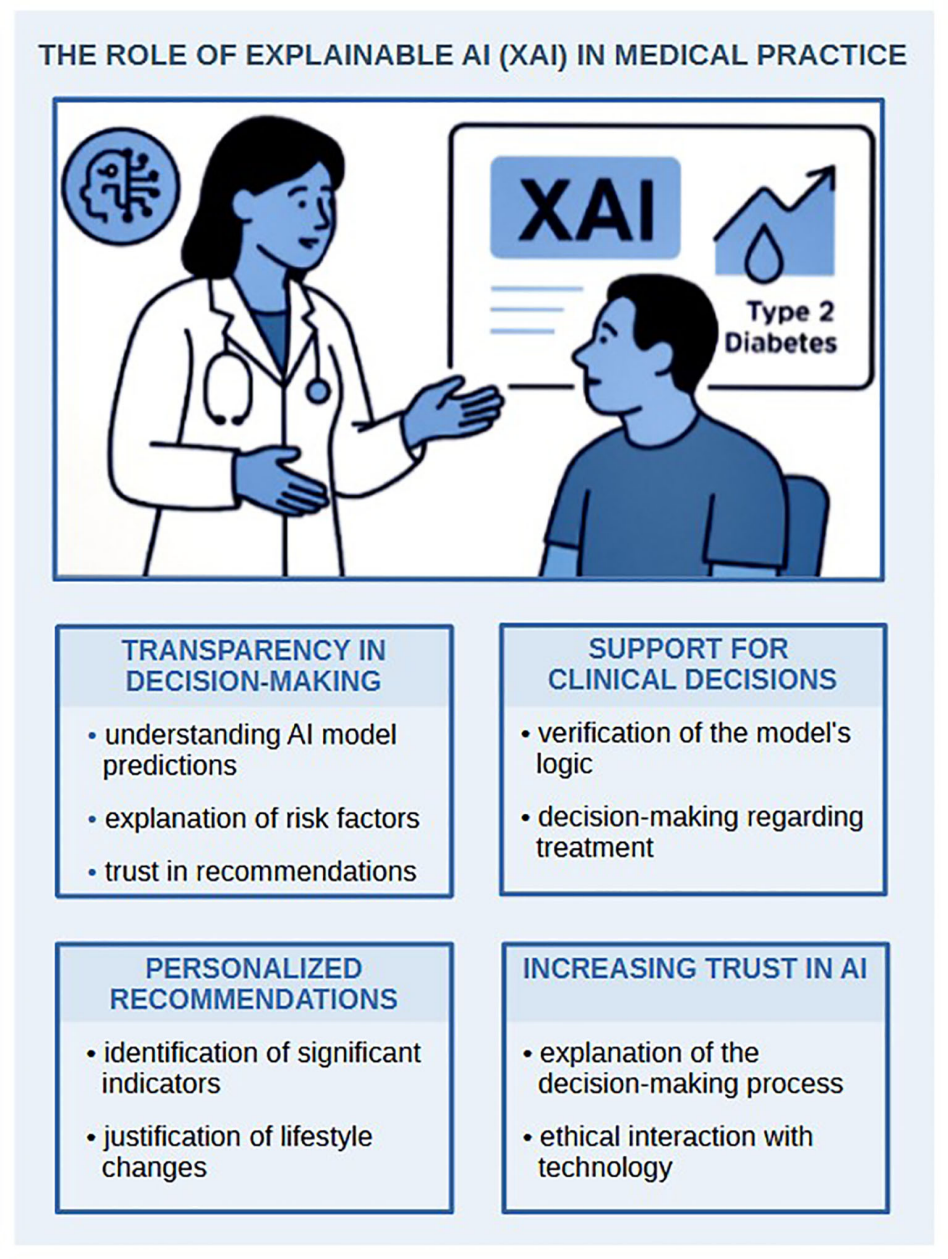
The role of explainable AI (XAI) in medical practice.

Together, these directions represent a paradigm shift: moving from universal treatment protocols toward dynamic, microbiome-oriented, and ethically personalized medicine. However, large-scale implementation of this model in clinical practice requires overcoming the challenges outlined above. Future research should prioritize:

- building open-access, well-annotated databases encompassing medical, metagenomic, and behavioral information for effective AI model training;
- developing explainable AI (XAI) models to enhance decision-making transparency, increase trust among clinicians and patients, and support ethical informed consent. We propose a block diagram that clearly illustrates the critical role of explainable AI (XAI) models (**Figure 3**);
- establishing regulatory and legal mechanisms to govern certification, liability, data protection, and algorithmic fairness;
- integrating intelligent models into telemedicine, which is particularly important for populations with limited healthcare access (165).
- developing standardized clinical protocols for integrating microbiota-based interventions and AI-driven platforms, such as DayTwo and Twin Health, into routine T2D management. This includes training clinicians to utilize AI tools for real-time monitoring and adjustment of personalized dietary and probiotic interventions, as well as incorporating these technologies into existing clinical guidelines to ensure seamless adoption in diverse healthcare settings.

To enhance the global applicability of findings on gut microbiota and T2D, there is a critical need for broader, multi-regional studies that investigate how various dietary patterns impact the interactions between microbiota and T2D. Regional diets, such as those high in corn in Latin America or high in red meat in Australia, likely affect the production of microbial metabolites, such as SCFA, as well as the overall composition of the microbiota. These factors can significantly impact the risk and progression of T2D. Our research group aims to address this gap in future studies by including populations from Latin America, Australia, and other underrepresented regions. These efforts will enhance the generalizability of our results and support the development of tailored, region-specific interventions for T2D management.

**Figure 4:**
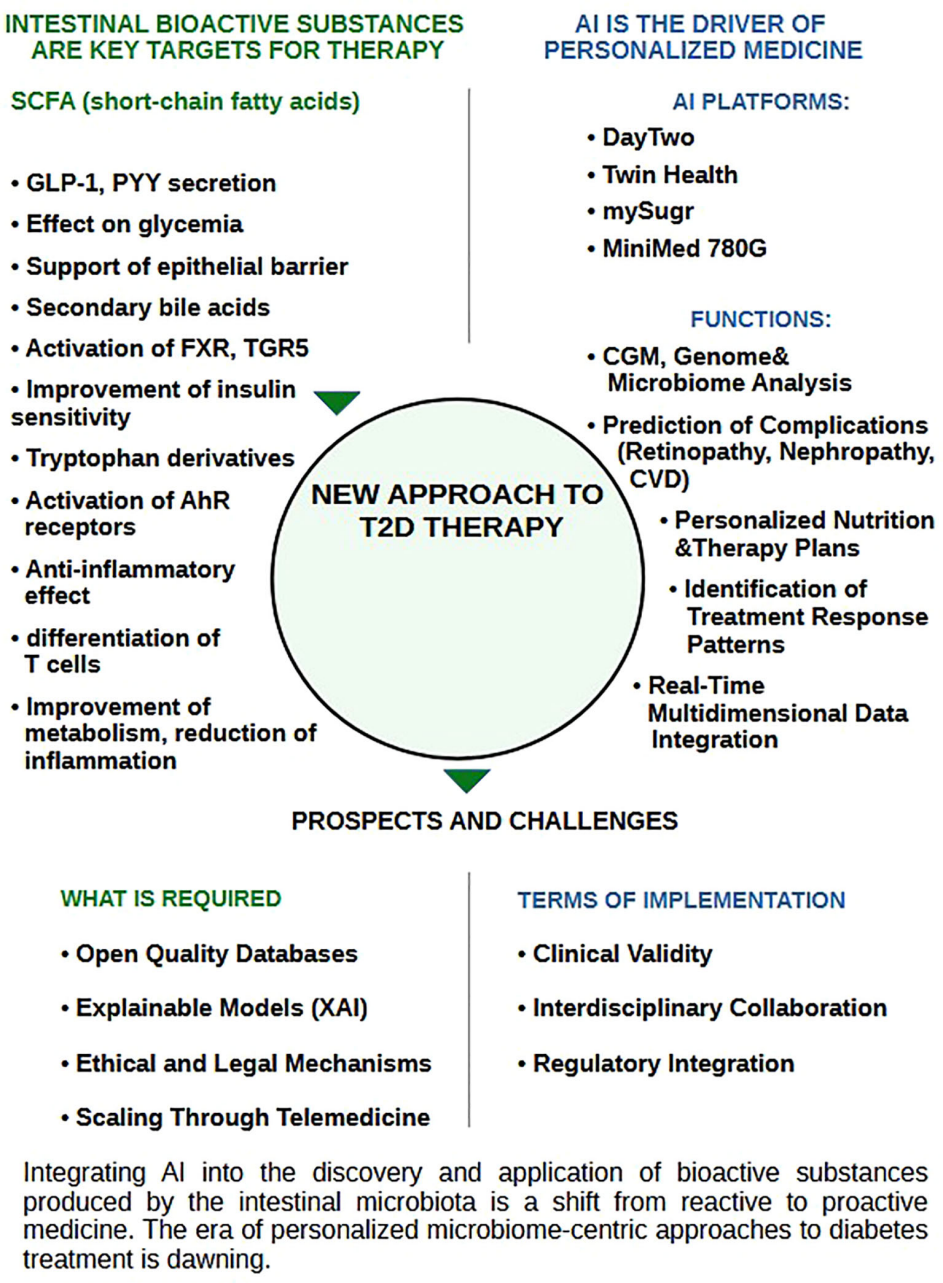
The synergy of gut-derived bioactive compounds and artificial intelligence tools - next-generation solutions for T2D management.

In summary, harnessing the synergy between bioactive microbiota components and AI not only optimizes T2D management but also drives the creation of a new digital medicine paradigm: a hybrid system in which advanced technologies enhance clinical expertise, respect patient autonomy, and provide the foundation for ethically and scientifically grounded microbiome-oriented therapy. Realizing this potential requires interdisciplinary consortia that unite experts in medicine, biology, ethics, and law. Only through such integrative efforts is it possible to establish a balanced implementation of innovations, where inclusivity, safety, humanity, and personalization define a new era in the fight against diabetes (**Figure 4**).
